# Exploring the relationship between social jetlag with gut microbial composition, diet and cardiometabolic health, in the ZOE PREDICT 1 cohort

**DOI:** 10.1007/s00394-023-03204-x

**Published:** 2023-08-02

**Authors:** Kate M. Bermingham, Sophie Stensrud, Francesco Asnicar, Ana M. Valdes, Paul W. Franks, Jonathan Wolf, George Hadjigeorgiou, Richard Davies, Tim D. Spector, Nicola Segata, Sarah E. Berry, Wendy L. Hall

**Affiliations:** 1 Department of Nutritional Sciences, King’s College London, London, UK; 2 ZOE Ltd, London, UK; 3 Department CIBIO, University of Trento, Trento, Italy; 4 School of Medicine, University of Nottingham, Nottingham, UK; 5 Nottingham NIHR Biomedical Research Centre, Nottingham, UK; 6 Department of Clinical Sciences, Lund University, Malmö, Sweden; 7 Department of Nutrition, Harvard TH Chan School of Public Health, Boston, MA USA; 8 Department of Twin Research and Genetic Epidemiology, King’s College London, London, UK

**Keywords:** Social jetlag, Gut microbiome, Diet

## Abstract

**Purpose:**

In this study, we explore the relationship between social jetlag (SJL), a parameter of circadian misalignment, and gut microbial composition, diet and cardiometabolic health in the ZOE PREDICT 1 cohort (NCT03479866).

**Methods:**

We assessed demographic, diet, cardiometabolic, stool metagenomics and postprandial metabolic measures (*n = *1002). We used self-reported habitual sleep (*n = *934) to calculate SJL (difference in mid-sleep time point of ≥ 1.5 h on week versus weekend days). We tested group differences (SJL vs no-SJL) in cardiometabolic markers and diet (ANCOVA) adjusting for sex, age, BMI, ethnicity, and socio-economic status. We performed comparisons of gut microbial composition using machine learning and association analyses on the species level genome bins present in at least 20% of the samples.

**Results:**

The SJL group (16%, *n = *145) had a greater proportion of males (39% vs 25%), shorter sleepers (average sleep* < *7 h; 5% vs 3%), and were younger (38.4 ± 11.3y vs 46.8 ± 11.7y) compared to the no-SJL group. SJL was associated with a higher relative abundance of 9 gut bacteria and lower abundance of 8 gut bacteria (*q < *0.2 and absolute Cohen’s effect size > 0.2), in part mediated by diet. SJL was associated with unfavourable diet quality (less healthful Plant-based Diet Index), higher intakes of potatoes and sugar-sweetened beverages, and lower intakes of fruits, and nuts, and slightly higher markers of inflammation (GlycA and IL-6) compared with no-SJL (*P < *0.05 adjusted for covariates); rendered non-significant after multiple testing adjustments.

**Conclusions:**

Novel associations between SJL and a more disadvantageous gut microbiome in a cohort of predominantly adequate sleepers highlight the potential implications of SJL for health.

**Supplementary Information:**

The online version contains supplementary material available at 10.1007/s00394-023-03204-x.

## Introduction

Sleep, along with diet and physical activity, is emerging as a potentially modifiable factor in improving health. Modern life with electric lights, blue-light emitting screens and work schedules disturb normal sleep patterns. Research links sleep disturbances with impaired health, [1] shift work is associated with an increased risk of cardiovascular disease (CVD), increased BMI, and metabolic disturbances [2, 3]. Emerging evidence suggests, beyond large circadian shifts in sleeping patterns such as shift work, smaller irregularities such as social jetlag (SJL) may be enough to affect health outcomes [4]. SJL is a pattern of sleep and wake times adjusted to workdays, and a shift in sleeping times on work-free days, which is more prevalent in individuals with late chronotypes (individuals who are biologically programmed for later bedtimes and wake times) [5]. SJL fluctuates with age and tends to peak in adolescents and young adults and tapers off with age [4, 6]. SJL is defined as a difference in mid-sleep point of 1 h or more on work versus work-free days, and it is estimated that SJL could affect more than 40% of the population [4].

Circadian misalignment may cause adverse changes in cardiovascular risk factors both directly and indirectly through diet; associations between SJL and poor diet, adiposity, and metabolic disturbances have been widely reported in specific populations such as adolescents and people with diabetes or metabolic syndrome [7–14]. For example, SJL has been associated with lower adherence to the Mediterranean diet and greater consumption of sugar-sweetened beverages in young people [15, 16], but it is unclear if these associations merely reflected reduced sleep duration in those with SJL. Analysis of a representative UK cohort of 19–64 year olds showed that those with SJL had lower fibre intake even if they had overall adequate sleep duration across the week [17]. There are several unconfirmed mechanisms whereby SJL could affect dietary choices, including disruption of appetite regulation and appetite hormone secretion [18] and altered central nervous system activity in reward centres [19].

Previous studies found associations between SJL and glycaemic dysregulation, lower high-density lipoprotein, and higher triglycerides, insulin, insulin resistance, adiposity, and metabolic syndrome [20–22]. Circadian misalignment caused by sleeping patterns that diverge from an individual’s circadian chronotype may cause dysregulation of metabolic and endocrine functions that fluctuate in tune with the circadian rhythm [22]. Studies show sleep disturbances impact inflammatory markers as well as an increase in oxidative stress which is a potential mechanism behind increased incidence of cardiovascular disease, [23] though further studies are needed to identify the exact mechanisms causing this association. Sleep deprivation causes important alterations in several intermediate biological mechanisms, such as the autonomic nervous system, endothelial function, insulin and glucose regulation, inflammation, and coagulation [1]. Elevated postprandial glycemia and lipemia are both associated with impaired health including oxidative stress, increased inflammation, beta cell dysfunction, lipoprotein remodelling and endothelial damage [24, 25], but there is a lack of postprandial data in relation to sleep duration and timing in large healthy populations. Therefore, investigating postprandial data in relation to SJL may further elucidate the relationship between sleep and cardiometabolic health.

A bidirectional relationship exists between sleep quality and the composition of the gut microbiome [26, 27]. Changes in gut microbiota composition also accompany several sleep disorders and pathologies with comorbid sleep disturbances, with a growing body of research implicating the microbiota–gut–brain axis in sleep physiology and behaviour [28]. Intestinal dysbiosis and circadian rhythm disruption are associated with similar diseases in humans including obesity, metabolic syndrome, and inflammatory bowel disease. Factors including diet and circadian misalignment are known to influence sleep and these effects may be mediated via the microbiota–gut–brain axis by the actions of circulating bacterial metabolites. Yet, it remains to be established whether some of the adverse effects associated with circadian disorganization in humans (e.g., in individuals with SJL) are related to dysbiosis, and to what extent this is mediated by poorer diet quality or specific dietary habits.

In the ZOE PREDICT cohort, an intervention study of diet–microbiome–cardiometabolic interactions, we observed associations between sleep disturbances and impaired postprandial glycaemic responses, [29] and between diet quality and gut microbiome [30], but associations between SJL and gut microbiome have not been explored. As previous literature suggests SJL is associated with poorer diet, then diet may be the mediating factor in any relationships revealed between SJL and gut microbiome. This study investigates (1) the relationship between SJL and the gut microbiome, dietary patterns, and fasting and postprandial markers of cardiometabolic health; and (2) potential mediating effects of diet in the relationship between SJL and the gut microbiome.

## Methods

### Study design and cohort

The ZOE PREDICT 1 study (NCT034798866) including data from 1002 monozygotic, dizygotic and non-twin healthy UK individuals aged 18–65 years was designed to quantify and predict individual variations in postprandial responses to standardised and free-living meals. Detailed descriptions of the study aims and protocol, including the health and lifestyle questionnaire used to capture self-reported sleep and other outcomes, can be found in the online protocol [31] and primary outcomes have previously been published [32]. The study was conducted in a real-world setting collecting in-clinic and at-home data including; genetics, metabolic markers, gut microbiome, exercise, sleep, order of meals, meal timing, meal composition, age, sex, and BMI. The PREDICT 1 study was a single-arm single-blinded intervention study conducted between June 2018 and May 2019. This is a secondary analysis of data from the clinical trial. We included data from *n = *934 individuals from the PREDICT cohort (Supplementary Fig. 1. CONSORT diagram) in this analysis. Ethical approval was obtained from the Research Ethics Committee and Integrated Research Application System (IRAS 236407), and the trial was run in accordance with the Declaration of Helsinki and Good Clinical Practice. There was patient and public involvement from TwinsUK participants during the design and conduct of the study.

### Sleep assessment

We calculated SJL using a self-reported questionnaire. Participants were asked questions for weekdays and weekend days (1) “At what time do you usually go to bed?” (2) “At what time do you usually wake up?”. We calculated SJL as the difference between midpoint sleep on weekends and weeknights in hours. It is recommended that SJL is analyzed both as a categorical and continuous variable [33], so we defined a difference of ≥ 1.5 h in the mid-sleep point between week (Sunday to Thursday) and weekend (Friday to Saturday) nights as the cut-off to differentiate between participants with SJL and those without SJL [4]. We also collected actigraphy data in PREDICT participants but we hypothesised the strict trial protocol (multiple fasted standardised breakfast meals and testing) influenced habitual sleep patterns on weekends. In the cohort, there were 3581 weekend days (Friday and Saturday) with actigraphy data, 92% of which a standardised meal was consumed on (median consumption time: 07:50am). For participants (*n = *290) with free-living weekend days (not following a trial protocol), 75% had one day which was not deemed reflective of habitual sleep patterns. Therefore, we chose subjective sleep data reporting ‘typical’ sleep patterns over the preceding month, over objective ‘real time’ actigraphy data to examine SJL.

Participants also completed the Pittsburgh Sleep Quality Index (PSQI) questionnaire, a self-report questionnaire that assesses sleep quality over a 1-month time interval. The PSQI is a validated tool measuring self-reported sleep quality and sleep disturbance on a scale from 0 to 21. A higher global PSQI score indicates worse sleep quality. A PSQI score of > 5 yields a sensitivity of 89.6% and a specificity of 86.5% [34]. We calculated habitual sleep duration using the average sleep duration across week and weekend days, short sleep was defined as < 7 h per night, average sleep was 7–9 h per night and long sleep was > 9 h per night [35]. We calculated chronotype using the self-reported question, “At what time do you typically go to sleep/wake up on weekends?”. We used the mid sleep time as a measure of chronotype [4], as a continuous variable later times were indicative of night-types and earlier times indicating morning-types.

### Gut microbiome sample collection and sequencing

Participants collected stool samples at home prior to the clinic visit using an EasySampler collection kit (ALPCO) and put samples into fecal collection tubes containing DNA/RNA Shield buffer (Zymo Research). A total of *n = *1001 stool samples were collected and processed for shotgun metagenomic sequencing. In brief, DNA was isolated by QIAGEN Genomic Services using the DNeasy 96 PowerSoil Pro kit and libraries prepared for 300-bp paired-end reads and sequenced using the Illumina NovaSeq 6000 platform with the S4 flowcell according to the manufacturer’s protocols. The microbiome methodology including DNA extraction and sequencing, metagenome quality and pre-processing, microbiome taxonomic and functional potential profiling and metagenomic assembly have been previously described [30].

### Blood samples

Participants attended a clinic day visit and consumed a standardised meal consisting of two high fat muffins and a milkshake. The total nutrient profile of the meal consisted of 890 kcal, 86 g of carbohydrates, 53 g of fat, 16 g of protein, and 2 g of fibre. We collected venous blood samples at 0, 15, 30, 60, 120, 180, 240, 270, 300 and 360 min. We analyzed the samples for postprandial glucose, insulin, serum C-peptide, triglyceride and lipid profile (NMR metabolomics). We calculated postprandial biomarkers including glucose 2 h incremental area under the curve (2hiAUC), insulin 2hiAUC and triglycerides 6hiAUC using the trapezoidal rule [36]. We measured GlycA at fasting, 4 h and 6 h postprandially using a high-throughput NMR metabolomics (Nightingale Health) 2016 panel [37].

### Glucose assessment

Participants wore a continuous glucose monitor (CGM) Freestyle Libre Pro (Abbott, Abbott Park, IL, US) for 14 days. The device was fitted on the clinic day by trained clinical practitioners to the participant’s non-dominant arm and measured interstitial glucose every 15 min. Due to the calibration of the CGMs, only data collected 12 h after fitting the device were used for analysis [36]. Outcomes measured by CGM were glucose variability (glucose coefficient of variation CV (%)), calculated by dividing glucose SD by mean glucose and time spent in range (TIR) based on optimised TIR targets (3.9–5.6 mmol/L) [38].

### Diet assessment

Participants completed the validated European Prospective Investigation into Cancer and Nutrition (EPIC) Food-Frequency Questionnaire (FFQ) which is used to measure habitual food and nutrient intakes over the past year. FETA software was used to calculate nutrient data and diet quality scores for the following indices; (1) Plant-based Diet Index (PDI), [39] (2) Healthful plant-based Diet Index (hPDI), (3) Unhealthful plant-based Diet Index (uPDI) positively scoring unhealthy plant-based foods and negatively for healthy plant-based foods, (4) Healthy eating index (HEI), [40] and (5) an alternate Mediterranean diet index (aMED) [41]. We excluded data if the total energy intake estimate calculated from the FFQ as a ratio of the subject’s estimated basal metabolic rate (determined by the Harris–Benedict equation) was more than 2 SD outside the population mean for this ratio (< 0.52 or > 2.58) or if more than ten items of the FFQ were left unanswered [32].

### Free-living diet data and diet habits variables

Participants consumed an ad libitum diet during 2–4 at-home study period days. We trained participants to accurately weigh and record ad libitum dietary intake using photographs, product barcodes, product-specific portion sizes, and digital scales. Data logged into the study app were uploaded onto a digital dashboard in real time. Nutrient values were obtained from the McCance and Widdowson Food and Nutrient database. For branded foods, nutrient information was collated from common supermarket websites. We excluded foods with unidentifiable names and free-living days per participant based on energy intake cut-offs (females; 500–5000 kcal, males; 500–8000 kcal). We calculated diet habit variables using the logged diet data. We defined an eating occasion (EO) as any occasion where a food or beverage was consumed that contained ≥ 50 kcal and was separated in time from the preceding and succeeding EO by 30 min. We defined a main meal as ≥ 400 kcal for females and ≥ 500 kcal for males. We defined snacks as eating occasions that were not main meals (females; 50–399 kcal, males 50–499 kcal). First and last eating occasion times were the time of day when the first and last eating occasion occurred (≥ 50 kcal). We calculated the fasting window as the difference between the last and first eating occasion. Eating midpoint was the midpoint in time between the first and last eating occasion. We calculated all diet habits variables for each free-living day and calculated mean free-living values.

### Appetite and mood scores

Participants reported their hunger levels on a visual analogue scale. App notifications appeared at *t = *0 (time of logging) and regular intervals (+ 0.5, + 1.5, + 2.5 h) following the logging of a breakfast, lunch or dinner meal. However, given the free-living conditions of the study, variable numbers of hunger ratings per day per participant occurred due to missed ratings. The app also prompted participants to report their hunger and anxiousness levels once per day at ~ 9 PM local time. The visual analog scale included the following question: “How have you been feeling generally over the whole day: How hungry/anxious have you felt?”. We calculated an average study hunger and anxiousness score using all ratings throughout the study period, in participants with ≥ 7 days of ratings.

### Statistical analysis

We analyzed the dataset using the statistical software package R (version 1.3.1093). We selected participants with complete data on sleep and wake-up times for both week and weekend days (*n = *945). We excluded participants when (1) sleep onset fell between 8 am and 5 pm (*n = *5), (2) sleep offset fell after 12 pm (*n = *1), (3) sleep duration was less than 2 h or more than 15 h, and (4) participants with negative SJL (*n = *5), since negative SJL occurs when mid-sleep point on weekdays are later than those on weekend days and it is recommended to look at negative and positive SJL separately [4]. The final cohort with self-reported sleep data after exclusions was *n = *934. We tested characteristics (age, sex, BMI, sleep duration, ethnicity, menopausal status, and education level) of both groups (SJL and no-SJL) using *χ*^2^ test and Kruskal test for categorical and continuous data, respectively. We tested differences in body composition, fasting blood biomarkers, postprandial biomarkers, lifestyle, microbiome, dietary intake and blood glucose measures (CGM derived) between both groups (SJL and no-SJL) using analysis of covariance (ANCOVA) adjusted for sex, age, BMI, ethnicity, and education status. We tested data for normality using Shapiro–Wilk and transformed where applicable using log or sqrt transformation. We defined a two-sided *P* value < 0.05 as the level of significance for interpretation of the statistical tests and correction for multiple testing (Benjamini–Hochberg, false discovery rate (FDR)) was applied. We made additional adjustments for habitual sleep duration (h), sleep quality, alcohol intake (g) and diet quality (hPDI). We did this to understand whether associations with SJL are due to sleep irregularities or short sleep duration. We examined correlations with difference between midpoint sleep on weekends and weeknights in hours (continuous variable) and dietary measures using the ‘ppcor’ package, adjusted for age, BMI, habitual sleep duration (h), sleep quality, alcohol intake (g) and diet quality (hPDI). We tested differences in relative abundances of species prevalent in ≥ 20% of both groups using the Mann–Whitney U test. We applied correction for multiple testing (Benjamini–Hochberg, FDR) and defined significance at FDR-corrected *P* value < 0.2 and absolute Cohen’s effect size > 0.2. We used the whole microbiome composition at the species-level genome bins (SGBs) only to train a machine learning model based on Random Forest to classify individuals into SJL and no-SJL classes. The evaluation was done on 100 bootstrap iterations with a 80/20 strategy for training and testing sets as previously described [30]. We created an age-matched subgroup of participants with and without SJL using the ‘*MatchIt*’ package. We selected participants from the two groups with overlapping age ranges and tested differences in relative abundances of species. We implemented the “*Mediation”* package to test the mediation effects of diet (indirect effects) on the total effect of SJL on microbiome species, adjusting for sex, age, BMI, ethnicity, education status and family-relatedness. We arcsine square root transformed relative abundances of gut microbiome species. We used linear mixed-effects models (“*lme4*” package in R) for both the mediator and outcome models. We created figures using GraphPad PRISM software.

## Results

### Participant characteristics

The total cohort was predominantly white (90%, *n = *837), 72% (*n = *676) female, had a mean BMI of 25.6 ± 5.0 kg/m^2^ and mean age of 46 ± 12 y (Table 1). Of the females, 50% were pre-menopausal, and 59% (*n = *552) of participants were educated to a university degree or higher. The mean difference between midpoint of sleep on week and weekend days was 0.83 ± 0.61 h (range; 0 to 3.5 h) (Supplementary Fig. 2). 16% (*n = *145) of participants were affected by SJL (≥ 1.5 h sleep midpoint difference on week vs weekend days). Participant characteristics stratified by SJL are displayed in Table 1. Only 3% (*n = *31) of total participants were classified as short sleepers (< 7 h per night) (SJL; 5% vs no-SJL; 3%), whilst 82% (*n = *764) had average sleep of 7–9 h per night and 15% (*n = *139) had more than 9 h per night. A greater proportion of participants with SJL were male (39% *vs* 25%). Those with SJL were younger, with a mean age 38 ± 11 y *vs* 47 ± 12 y and there was a higher proportion of pre-menopausal females (72 vs 46%).

**Table 1:** Participant characteristics from PREDICT 1 (18–65 y) according to social jetlag status

	All participants (*n = *934)	No social jetlag (*n = *789)	Social jetlag (*n = *145)	*P* value
Sex, *n* (%)				
Females	676 (72)	588 (75)	88 (61)	
Males	258 (28)	201 (25)	57 (39)	< 0.001
Age (y)	45.5 (12.0)	46.8 (11.7)	38.4 (11.3)	< 0.001
Body mass index (kg/m^2^)	25.6 (5.0)	25.6 (4.9)	25.4 (5.7)	0.156
Sleep duration weekdays (h)	8.1 (0.9)	8.1 (0.9)	7.8 (0.9)	< 0.001
Sleep duration weekend days (h)	8.7 (1.0)	8.6 (0.9)	9.0 (1.1)	< 0.001
Habitual sleep duration (h)	8.4 (0.8)	8.4 (0.8)	8.4 (0.8)	0.560
Ethnicity, *n* (%)				
Asian or Asian British	13 (1)	9 (1)	4 (3)	
Black, African, Caribbean or Black British	15 (2)	11 (1)	4 (3)	
Mixed or multiple ethic groups	23 (3)	21 (3)	2 (1)	
Other ethnic groups	10 (1)	5 (1)	5 (3)	
White	837 (90)	711 (90)	126 (87)	
Unknown	36 (4)	32 (4)	4 (3)	0.014
Menopausal status, *n* (%)				
Perimenopausal	49 (7)	46 (8)	3 (3)	
Post-menopausal	190 (28)	183 (31)	7 (8)	
Pre-menopausal	335 (50)	273 (46)	63 (72)	
Unknown	102 (15)	86 (15)	15 (17)	< 0.001
Education level, *n* (%)				
Below university	345 (37)	304 (39)	41 (28)	
University degree or higher	552 (59)	452 (57)	100 (69)	
Unknown	37 (4)	33 (4)	4 (3)	0.031

*P* value < 0.05 shows significant statistical difference

### Gut microbiome

**Fig. 1:**
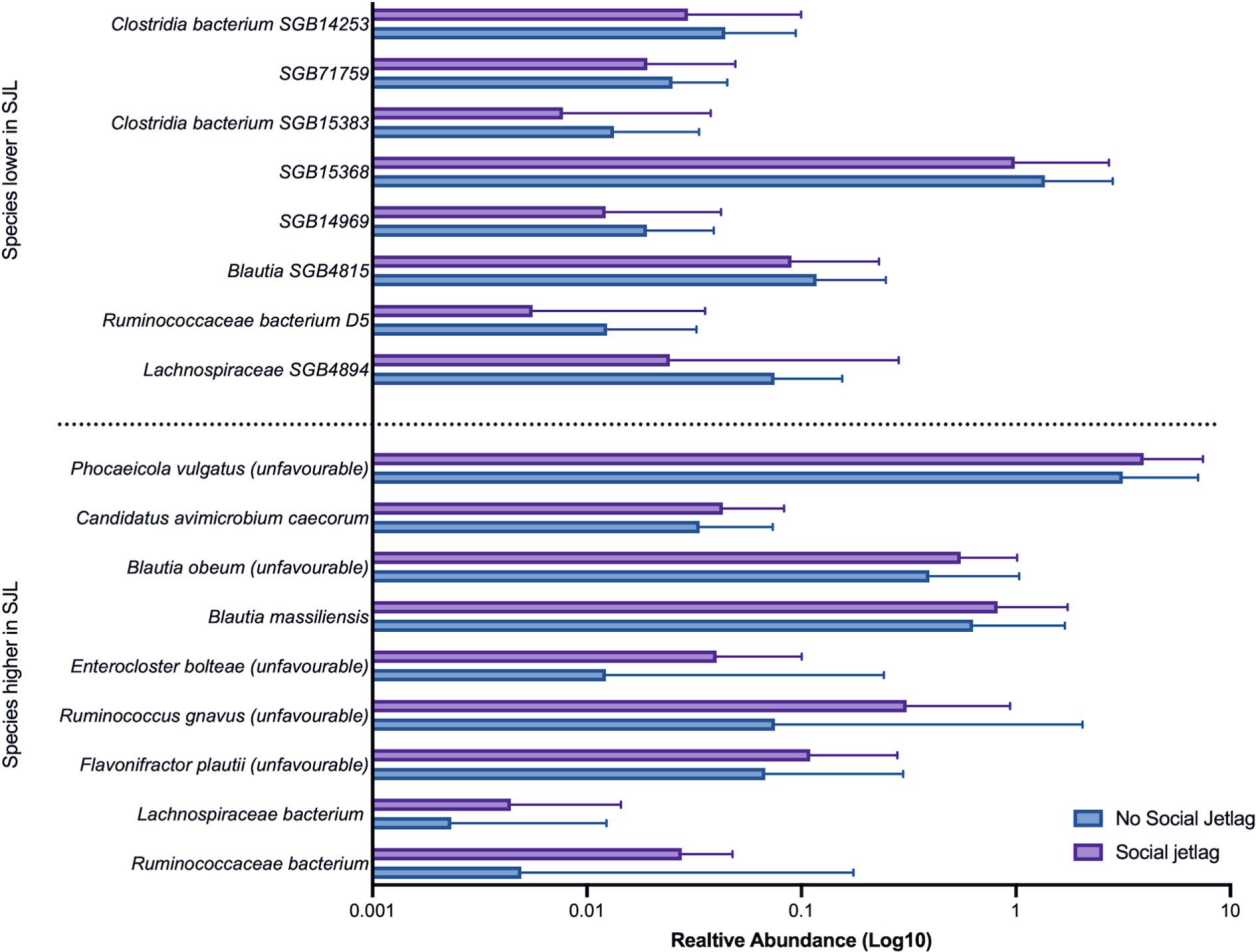
Gut microbiome species that are at least 20% prevalent and differentially abundant in the social jetlag (SJL) versus no-social jetlag groups, showing a significant FDR-corrected *P* value < 0.2

The relative abundances of bacterial species prevalent in both groups (≥ 20%) were differentially abundant according to SJL status (Supplementary Table 1). Seventeen species were significantly different between the groups (Mann–Whitney U *q < *0.2 and absolute Cohen’s effect size > 0.2) (Fig. 1). Nine species had greater abundances in those with SJL and 8 were greater in those with no-SJL. These species included previously characterised species as well as some newly uncharacterised species (*Lachnospiraceae* unclassified SGB4894, SGB14969, and SGB71759) captured by integrating genomic information of uncultured species. We also examined whether there were species prevalent (≥ 20%) in one group and not in the other; three species (*Clostridia* bacterium SGB14263, *Clostridia* bacterium SGB3940, *Peptococcaceae* bacterium GB49168) had marked differences in prevalences (absolute difference between groups; 18.1%, 12.1%, and 11.5%, respectively). These three species were prevalent (≥ 20%) in participants without SJL only. Although abundances and prevalence of individual species differed, the whole microbiome was not able to discriminate between those with SJL versus no-SJL (median AUC = 0.572). To determine whether these associations were independent of age, we examined an age-matched subgroup, finding fifteen species were differentially abundant according to SJL status. Thirteen out of the seventeen species differentially abundant according to SJL status in the original cohort remained significant and two new species were identified (SGB1877 and SGB15154) (Supplementary Table 2).

### SJL–diet–microbiome relationship

As diet is a known driver of microbiome composition, we explored the mediation effect of diet with SJL and microbiome composition (including the 13 differentially abundant species identified above consistent in the total and age-matched cohorts). Diet quality in part mediated the relationship between SJL and two microbiome species, SGB71759 and *Ruminococcaceae* bacterium (Supplementary Table 3). The mediation analysis revealed that diet quality acted as a potential partial mediator in the association between SJL and SGB71759 (proportion mediation; 9%, *P* = 0.036). *Ruminococcaceae* bacterium was in part mediated by diet but the proportion mediated was smaller (4%, *P < *0.05). Intakes of nuts was the most strongly associated food with SJL and a marker of healthful food choice. We, therefore, also looked at mediating effects of this food; interestingly, we found nut intakes mediated the relationship between SJL and the same microbiome species (proportion mediation; 15%, *P* = 0.01 and 5%, *P* = 0.02 for SGB71759 and *Ruminococcaceae* bacterium respectively) but to a greater extent. Further*,* we identified two additional new species, *Flavonifractor plautii* (proportion mediation; 9%, *P* = 0.02) and *Clostridia* bacterium SGB14253 (proportion mediation; 12%, *P* = 0.036) mediated by nuts*.*

### Associations between SJL and diet

**Fig. 2:**
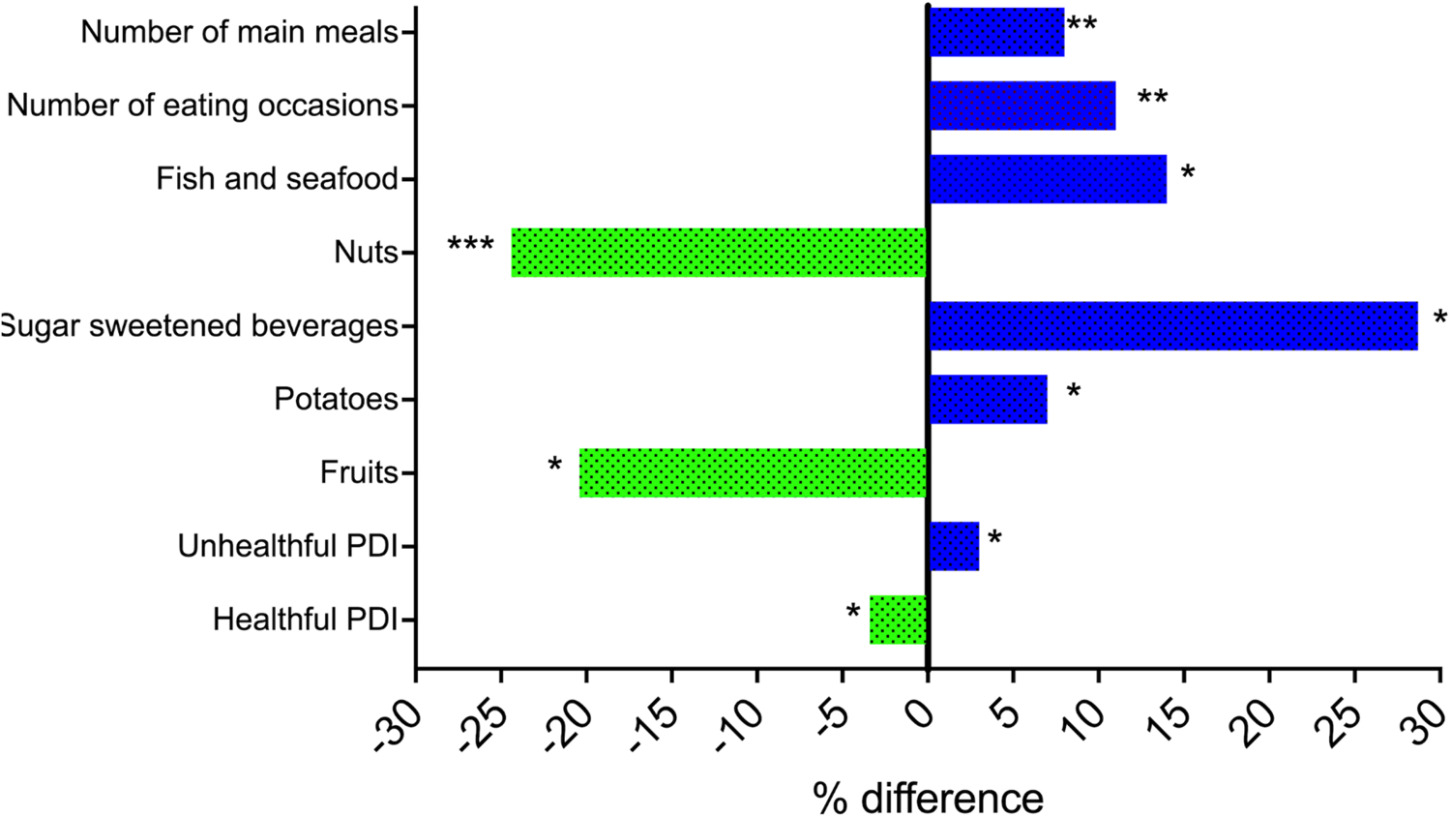
Selection of diet and habit variables that were different between participants with SJL and no-SJL (% difference). Analyses were adjusted for sex, age, BMI, ethnicity, and education

Differences in dietary quality indices, food groups and nutrient intakes are presented in Supplementary Table 4 and Fig. 2 (all adjusted for sex, age, BMI, ethnicity, and education). Participants with SJL had slightly poorer diet quality (hPDI; 58 ± 7 and uPDI; 61 ± 7) compared to no-SJL (hPDI; 60 ± 7 and uPDI: 59 ± 7). SJL was also associated with higher intakes of fish and seafood (including oily fish, fresh fish, seafood, fish fingers and fried fish) (52.2 g ± 41.8 vs 45.0 g ± 35.8), sugar sweetened beverages (87.7 g ± 160 vs 62.5 g ± 119) and potatoes (including boiled, roast, fried, crisps, potato salad) (76.3 g ± 55.9 vs 71.0 g ± 48.8) compared with no-SJL (*P < *0.05 for all, but after FDR correction no significance remained). Conversely, no-SJL was associated with a higher intake of healthful foods including fruits (211 g ± 147 vs 175 g ± 135) and nuts (18.0 g ± 20.4 vs 14.5 g ± 17.9). After adjustments for habitual sleep duration and sleep quality the differences between the SJL group and no-SJL remained, apart for fruit when adjusted for sleep quality. When we examined correlations between the difference between midpoint sleep on weekends and weeknights in hours (continuous variable) and dietary measures, intake of nuts was inversely correlated with SJL (*r*;  − 0.13, *P < *0.05) (Supplementary Table 4).

We also examined timing-related free-living dietary habits including number of eating occasions, cumulative number of main meals, eating window (hrs), first and last eating occasion (clock time), eating midpoint (midpoint between first and last eating occasions) and chronotype (Supplementary Table 5). Participants with SJL had fewer eating occasions (4.7 ± 1.2 *vs* 5.2 ± 1.3) and fewer main meals (2.4 ± 0.8 *vs* 2.6 ± 0.8) than those with no-SJL, although the effect size was small (*P < *0.001). Participants with SJL included more late type chronotypes, defined by a later mid-sleep time on weekend days (04:30 am (IQR; 04:00, 05:15) *vs* 03:30 am (IQR; 03:00, 04:00)). They also had a delayed time of their first main meal. No other associations were evident with the other timing-related free living dietary habit variables.

### Markers of health

We found no differences between the SJL groups for body composition (Table 2). We observed higher concentrations of fasting GlycA (measure of systemic inflammation) (1.35 mmol/L ± 0.19 *vs* 1.32 mmol/L ± 0.18) and IL-6 (0.95 ng/L ± 2.62 *vs* 0.69 ng/L ± 0.65) in the SJL group, compared to the no-SJL group (*P < *0.05 for all), although the effect size was small. Self-reported anxiousness was also higher in those with SJL (31.4 ± 16.2 vs 26.0 ± 15.3). Significance was lost after adjustment for multiple testing (FDR-adjustment). We found no other differences in fasting or postprandial blood biomarkers or 24 h glucose data (measured by CGM), nor did the results change after adjustment for average sleep duration, sleep quality, diet quality or alcohol intakes (Table 2). Next, we investigated associations between SJL and GlycA according to age, sex and menopausal status. This effect was assessed by including an interaction term determining whether the relationship between SJL and GlycA changes depending on the value of age, sex or menopause (separate models).

**Table 2:** Markers of health from the ZOE PREDICT 1 cohort stratified by social jetlag status

	Total (*n = *934)			Social jetlag (*n = *145)			No social jetlag (*n = *789)			*P* value	Adjusted; habitual sleep duration	Adjusted; sleep quality	Adjusted; diet quality (hPDI)	Adjusted; alcohol intake	Adjusted; all covariates plus multiple testing (FDR)
	*n*	mean	SD	*n*	mean	SD	*n*	mean	SD		*P* value	*P* value	*P* value	*P* value	*P* value
Waist to hip ratio (cm)	933	0.84	0.08	145	0.85	0.09	788	0.84	0.08	0.284	0.280	0.652	0.269	0.182	0.827
Systolic BP (mm/Hg)	920	124	14.7	142	122	12.1	778	125	15.1	0.972	0.987	0.865	0.993	0.933	0.918
Diastolic BP (mm/Hg)	920	75.8	10.2	142	74.4	10.1	778	76	10.2	0.925	0.907	0.955	0.806	0.879	0.918
Fasting blood biomarkers															
Glucose (mmol/L)	933	4.95	0.48	144	4.89	0.49	789	4.97	0.48	0.570	0.577	0.976	0.740	0.763	0.917
Triglycerides (mmol/L)	934	1.06	0.54	145	1.10	0.51	789	1.05	0.54	0.091	0.093	0.103	0.057	0.040	0.531
Insulin (mIU/L)	934	6.13	4.06	145	6.56	4.41	789	6.05	3.99	0.133	0.133	0.112	0.177	0.095	0.531
GlycA (mmol/L)	933	1.33	0.18	145	1.35	0.19	788	1.32	0.18	0.042	0.043	0.039	0.064	0.029	0.531
IL-6 (ng/L)	934	0.73	1.19	145	0.95	2.62	789	0.69	0.65	0.027	0.028	0.027	0.045	0.035	0.531
Cholesterol (mmol/L)	934	4.92	0.98	145	4.77	0.96	789	4.95	0.97	0.505	0.511	0.777	0.387	0.325	0.827
LDL (mmol/L)	919	2.92	0.79	143	2.83	0.81	776	2.94	0.79	0.378	0.381	0.827	0.265	0.213	0.827
HbA1c (%)	931	5.47	0.28	144	5.41	0.28	787	5.48	0.28	0.582	0.595	0.397	0.433	0.595	0.827
HOMA IR	934	1.38	1.05	145	1.49	1.24	789	1.37	1.01	0.119	0.120	0.094	0.147	0.095	0.531
Liver Fat Probability score	864	0.17	0.14	129	0.17	0.15	735	0.17	0.14	0.625	0.621	0.617	0.631	0.583	0.827
Postprandial biomarkers															
Glucose (2 h iAUC)	843	7465	5048	123	6910	4757	720	7560	50,923	0.572	0.578	0.835	0.473	0.397	0.827
Insulin (2 h iAUC)	842	268,592	172,713	123	275,912	217,685	719	267,340	163,943	0.631	0.612	0.387	0.646	0.854	0.827
Triglyceride (6 h iAUC)	758	10,404	7630	111	11,098	7810	647	10,285	7599	0.157	0.154	0.517	0.239	0.256	0.827
GlycA (6 h rise, mmol/L)	893	-0.03	0.05	139	-0.04	0.04	754	-0.03	0.05	0.837	0.840	0.533	0.818	0.866	0.827
Microbiome															
Richness (number of species)	934	118	17.3	145	118	18.2	789	118	17.1	0.568	0.567	0.352	0.373	0.286	0.698
Diversity (Shannon Index)	934	3.24	0.35	145	3.22	0.36	789	3.25	0.35	0.867	0.850	0.716	0.876	0.790	0.827
CGM glucose measures															
Glucose variability (CV, %)	759	16.4	4.29	114	15.7	3.63	645	16.5	4.38	0.611	0.609	0.724	0.386	0.445	0.827
Optimised TIR (%)	759	68.9	16.8	114	70.5	16.3	645	68.7	16.9	0.240	0.239	0.339	0.216	0.217	0.827
Satiety and mood															
Average hunger (study duration)	909	38.6	13.5	144	41.4	14.2	765	38	13.3	0.521	0.523	0.082	0.608	0.653	0.531
Average anxiousness	616	26.9	15.5	102	31.4	16.2	514	26.0	15.3	0.050	0.050	0.127	0.096	0.120	0.698

*hPDI* healthy plant-based diet index; *BP* blood pressure; *LDL* low-density lipoprotein; *HDL* high-density lipoprotein; *TIR* time-in-range

ANCOVA; *P* values adjusted for sex, age, BMI, ethnicity, SES, except for ASCVD risk (age and sex removed) and for height, weight, visceral fat and waist to hip ratio, BMI was removed

Overall, participants with SJL had higher concentrations of GlycA, but the effect of SJL depended on the participant’s sex (*P* = 0.035) and age (*P* = 0.044). The SJL x sex interaction remained significant after adjustment for covariates (age, BMI, ethnicity and education), showing males with SJL had higher inflammation compared to all females (SJL (diff; − 0.05 mmol/L, *P* = 0.001) and no-SJL (diff; − 0.04 mmol/L, *P < *0.001)) and males with no-SJL (diff; − 0.03 mmol/L, *P* = 0.026). There was also a SJL x menopause status interaction whereby perimenopausal women were more susceptible to SJL-related inflammation compared to pre-menopausal females (*P* = 0.013) or post-menopausal females (*P* = 0.044).

## Discussion

To our knowledge this is the first study finding multiple associations between SJL and diet quality, diet habits, inflammation, and gut microbial composition within a single cohort. Our findings suggest that a small degree of circadian misalignment is associated with diet and non-diet-mediated gut dysbiosis that may increase risk of chronic non-communicable diseases.

Given the growing importance of microbiota in human diseases linked with disturbed sleep and circadian rhythms, we investigated the role of chronic circadian misalignment on the microbiome. Although we did not find the overall microbiome composition to be predictive of SJL *vs.* no-SJL, single-species associations were found to be differentially abundant between SJL and no-SJL and were independent of age. Using the 13 species consistent in the total and age-matched sub-cohorts, three out of the six species more abundant in the SJL group have previously been identified as “unfavourable” and associated with dietary quality and diversity, [30] while the other three have an average rank closer to “unfavourable” species. Seven species showed greater abundance in those with no-SJL and were largely from yet-to-be-characterized species, highlighting the importance of this new approach integrating genomic information of uncultured species that enables deeper investigations of microbiome samples. Due to the complex relationship between sleep, diet and the microbiome, we explored the potential mediating effect of diet on the unfavourable relationship between SJL and the gut microbiome. Diet in part mediated the relationship between microbiome species in those with SJL, suggesting that modifiable dietary factors may play a role in the unfavourable variation in gut microbiome observed with circadian misalignment.

Animal studies demonstrate that circadian disorganization impacts the intestinal microbiota [42]; repeated phase shifts have been shown to alter the intestinal microbiota composition in circadian disrupted mice fed a high-fat, high-sugar diet but not in mice that were fed a standard chow diet [26]. A review by Parkar and colleagues [43] suggests that there is an interdependency between host circadian systems and gut microbiome, where imbalances and interactions between them have consequences for host metabolism. Metabolites produced by the microbiome have multiple functions, one proposed mechanism is that disrupted sleep and impaired diet quality can lead to gut dysbiosis and lower the production of butyrate, which will affect substrate oxidation and energy regulation in the host, impacting the risk of obesity and metabolic diseases [43]. A reverse causal relationship may also exist where gut dysbiosis causes sleep disturbances via the gut brain axis [44]. However, SJL-associated sleep restrictions are imposed by external (social or work) obligations so associations observed in this study are more likely to reflect  a direct and indirect effect of sleep timing, via diet, on species profiles.

The significant differences observed in diet quality between the SJL and no-SJL groups are consistent with previous findings that people with SJL consume more high energy-dense foods and less fibre, fruit, and vegetables [15, 17, 45–50]. Higher ghrelin levels have been observed in the biological evening than biological morning and circadian misalignment increased participants’ appetite for energy dense foods [51]. However, a relationship between SJL and hunger was not observed in this study, possibly related to the timing of appetite scores. Additionally, changes in diet habits due to circadian misalignments may also contribute to SJL-diet-health interactions [47, 48, 51, 52]. In this study, individuals with SJL had delayed timing for the first main meal and a greater tendency to have a late chronotypes, which together may contribute toward the unfavourable associations observed with diet.

Slightly higher concentrations of the inflammatory biomarkers, GlycA and IL-6, were observed in those with SJL, although this did not remain significant following adjustment for multiple testing. Chronic low-grade inflammation is an underlying factor in non-communicable chronic diseases. Whilst previous studies have found associations between SJL and markers of inflammation in metabolically unhealthy people with obesity and individuals living with diabetes [15], our results suggest that these associations may be present at a subclinical level in a healthy cohort, and certainly not in the region of the effect sizes (0.24 mmol/L) associated with increased risk of future cardiovascular events [37]. Interestingly, ASCVD 10 y risk was different between SJL and no-SJL only after adjustment for weekday sleep duration, suggesting that there is a protective effect of higher weekday sleep. This aligns with the belief that getting sufficient average sleep may potentially mitigate some of the detrimental effects of circadian misalignment [14]. The associations between SJL and metabolic risk factors such as fasting insulin, glucose, triglycerides, insulin resistance and lipid profiles are well established. However, most previous studies have often been in populations with obesity, diabetes, or metabolic syndrome. [6, 10, 11] Our study found no significant differences in fasting or postprandial biomarkers, which may be because the cohort consisted mainly of healthy, lean, adequate sleepers; the PREDICT 1 total cohort are healthier than a UK-representative cohort and the proportion of short sleepers is also lower than the national average [54].

A recent review suggests that about 70% of students and working adults have SJL of ≥ 1 h, and about 50% have a ≥ 2 h SJL [55]. In comparison, our cohort has only 16% having SJL of ≥ 1.5 h, and a very low prevalence of inadequate sleepers with only 3% sleeping < 7 h on average, possibly related to the demographic composition of the PREDICT 1 cohort. Previous studies have found that circadian rhythm is highly individualised, and that SJL is more prevalent in late chronotypes [56]. SJL is also often age-dependent, with an increase in adolescence and young adulthood [56]. These trends were confirmed in our data with the SJL group being younger in age and having a later chronotype. More longitudinal studies on the long-term effects of SJL are needed. According to Roenneberg et al. it is unlikely that the poor health outcomes associated with SJL are due to having a different sleeping pattern on weekdays and weekends or being a late chronotype, and more likely to be related to consequences of having to adjust to schedules unsuitable for one’s individual chronotype [4]. Previous studies have struggled to establish whether the detrimental effects associated with SJL are related to circadian misalignment or sleep loss [57]. The differences we have found suggest there may be an association of SJL with unfavourable health even under conditions of sufficient sleep.

Our study is unique in that it includes a deeply phenotyped cohort at a granularity greater than any other SJL study, allowing multiple comparisons. Some limitations of this study include (1) our cross-sectional statistical design meaning we cannot determine directionality of causality, (2) no information on shift work or employment status, (3) our use of subjective sleep measures based on habitual sleep, and calculations based on time in bed rather than time sleeping, as data on sleep latency was lacking, (4) our use of week versus weekend days as we did not have information on work versus work-free days, (5) inability to determine current sleep medication use. Although we selected subjective sleep data over objective actigraphy data due to the impact of the strict trial protocol (multiple fasted standardised meals and testing) on habitual sleep patterns, Tsereteli et al. [29] previously reported similar patterns of average sleep in the PREDICT 1 cohort using accelerometer derived sleep data compared to our self-reported average sleep levels in this study.

In conclusion, this study suggests that minor circadian misalignment in the form of SJL is associated with distinct gut microbiome species, which may be partly mediated through dietary differences. As circadian rhythm is individualized, adjusting sleep to one’s biological clock is preferable but not always achievable in the context of social timing. Guidance on achieving sufficient sleep with consistent sleep–wake timing and maintaining a healthy diet are potential modifiable lifestyle changes which may have potential to reduce future risk of disease. These findings raise the possibility that socially-imposed chronic circadian rhythm disturbance caused by sleep–wake shifts could influence the human gut microbiota, which may guide possible microbial therapies for clinical intervention in sleep related diseases such as obesity, diabetes, cardiovascular disorders, and cognitive impairments. Further studies including a broader cohort, objective sleep data, and longitudinal data collection to determine causality as well as long-term implications of SJL are needed to further understand the effect of SJL on health outcomes.

### Supplementary Information

Below is the link to the electronic supplementary material.

Supplementary file1 (DOCX 1183 KB)

Supplementary file2 (XLSX 145 KB)

## Data Availability

The data used for analysis in this study are held by the Department of Twin Research at King’s College London and access can be requested from https://twinsuk.ac.uk/resources-for-researchers/access-our-data/ to allow for anonymisation and compliance with GDPR standards.
