# Supplementary material for: Exploring the relationship between social jetlag with gut microbial composition, diet and cardiometabolic health, in the ZOE PREDICT 1 cohort
Source: Eur J Nutr. 2023 Aug 2;62(8):3135–47. doi: 10.1007/s00394-023-03204-x (PMC10611873; doi:10.1007/s00394-023-03204-x)
Supplement: Supplementary file 1 — Supplementary file1 (DOCX 1183 KB) [file 394_2023_3204_MOESM1_ESM.docx]

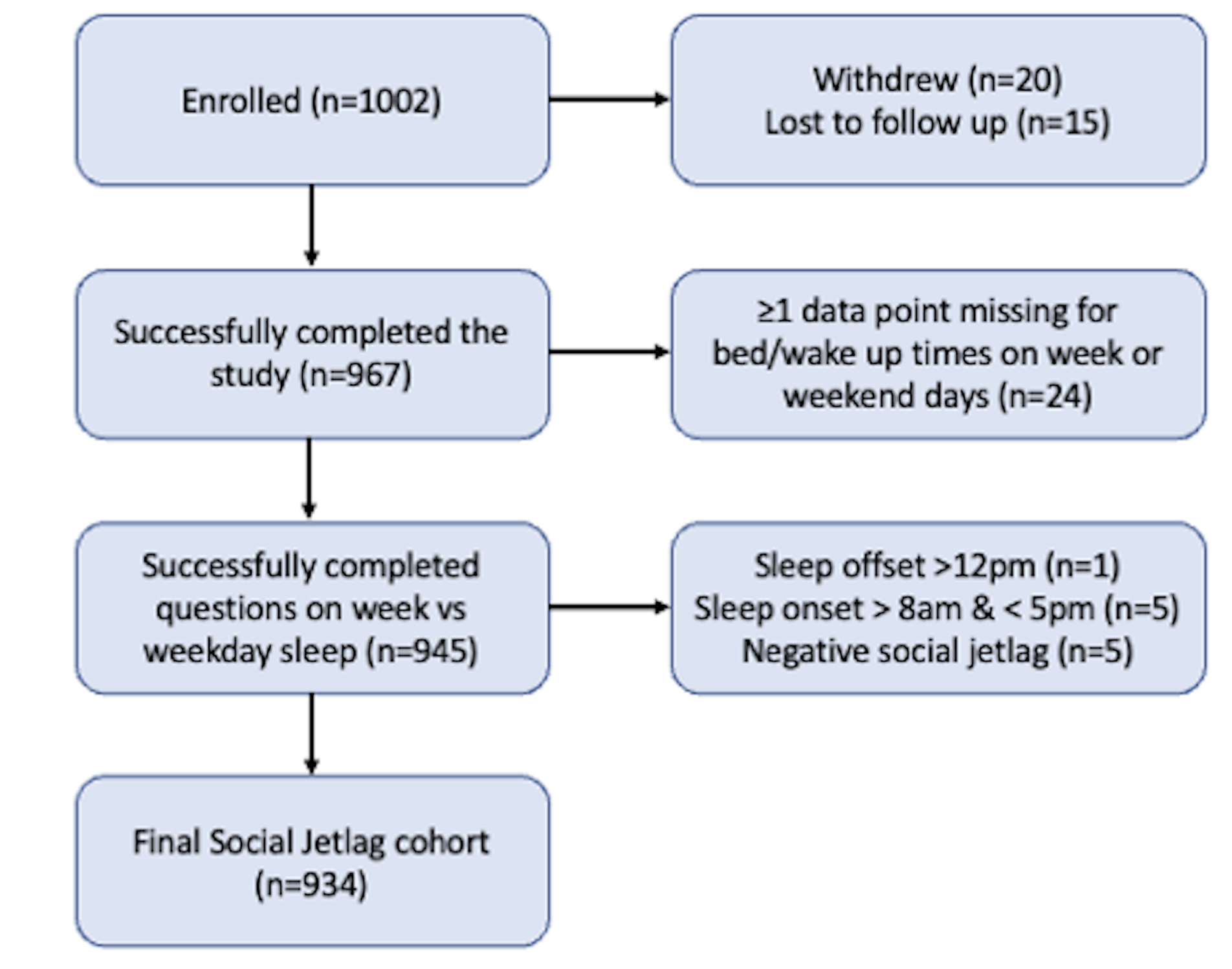


**Supplementary Fig 1** CONSORT diagram for the PREDICT 1 UK study

**
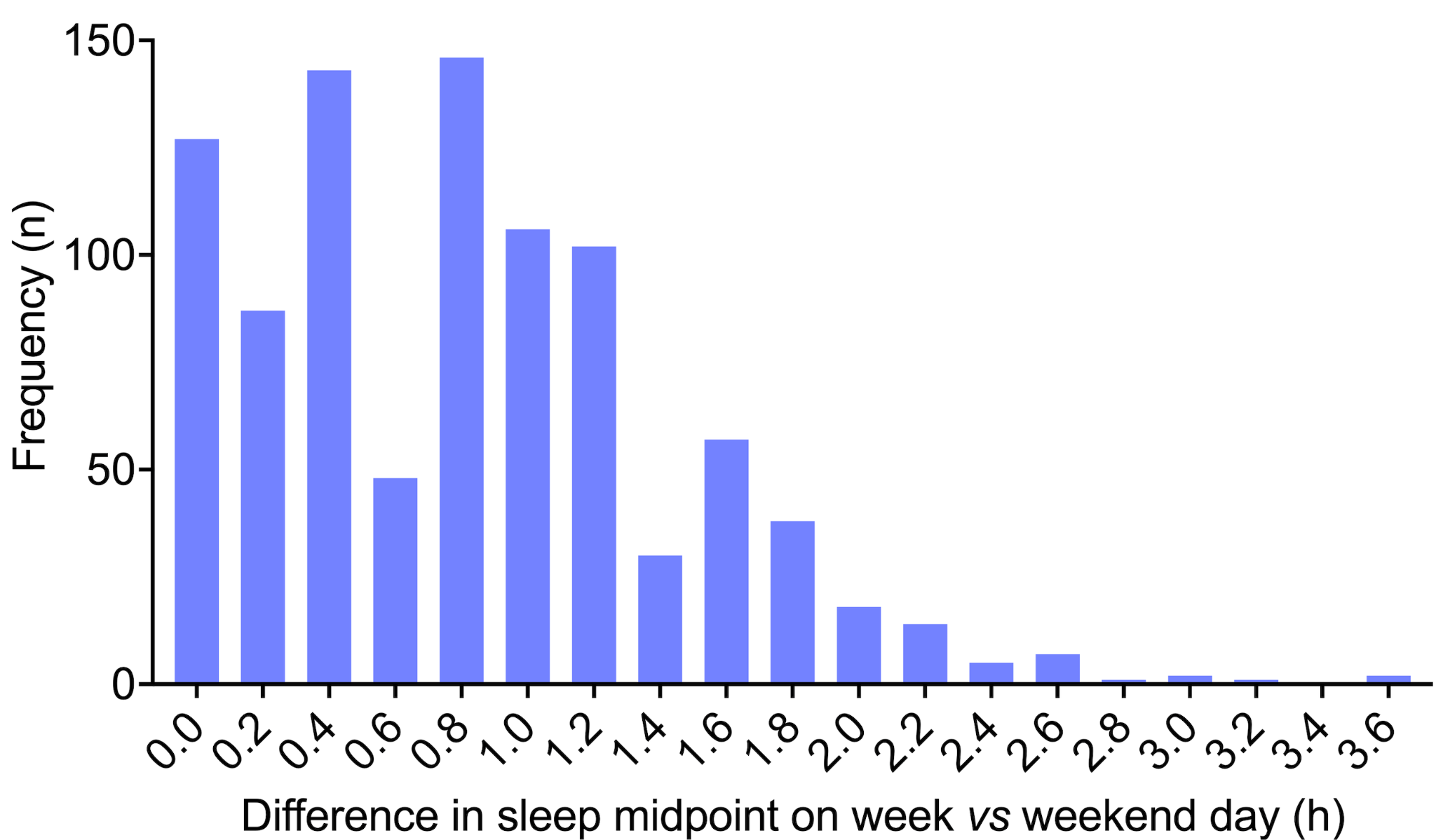
**

**Supplementary Fig 2** Distribution of social jetlag in the PREDICT population
